# Small secreted proteins and exocytosis regulators: do they go along?

**DOI:** 10.1080/15592324.2022.2163340

**Published:** 2023-02-12

**Authors:** Tamara Pečenková, Martin Potocký

**Affiliations:** a Laboratory of Cell Biology, Institute of Experimental Botany of the Czech Academy of Sciences, Prague, Czech Republic; b Department of Experimental Plant Biology, Faculty of Science, Charles University, Prague, Czech Republic

**Keywords:** PR1, CLE, co-expression, secretion, SNARE, exocyst

## Abstract

Small secreted proteins play an important role in plant development, as well as in reactions to changes in the environment. In *Arabidopsis thaliana*, they are predominantly members of highly expanded families, such as the pathogenesis-related (PR) 1-like protein family, whose most studied member PR1 is involved in plant defense responses by a so far unknown mechanism, or Clavata3/Endosperm Surrounding Region (CLE) protein family, whose members’ functions in the development are well described. Our survey of the existing literature for the two families showed a lack of details on their localization, trafficking, and exocytosis. Therefore, in order to uncover the modes of their secretion, we tested the hypothesis that a direct link between the secreted cargoes and the secretion regulators such as Rab GTPases, SNAREs, and exocyst subunits could be established using *in silico* co-expression and clustering approaches. We employed several independent techniques to uncover that only weak co-expression links could be found for limited numbers of secreted cargoes and regulators. We propose that there might be particular spatio-temporal requirements for PR1 and CLE proteins to be synthesized and secreted, and efforts to experimentally cover these discrepancies should be invested along with functional studies.

## Introduction

Plants are sessile organisms that can neither escape nor fight back upon a threat, and yet, to avoid tissue damage and energy losses, they can rapidly adjust to a changeable surroundings. Even at the exact moment, environmental conditions can be extremely different for distant parts of plant bodies. On top of that, plants maintain a constitutive supply of the plasma membrane lipids and proteins, cell wall building blocks, and extracellular space content via secretion [rev. in^1,2^]. For all these demands to be fulfilled, proper and precise regulation of secretory pathways is a prerequisite. The secretion regulators of plants are conserved and homologous to those of other multicellular organisms [rev. in^3–5^]. However, genomes of flowering plants underwent amplifications, also leading to the multiplication of regulatory combinations, probably in proportion to the complexity of the secretome [e. g.^6^].

In the model plant *A. thaliana*, various small secreted proteins (SSPs) have been discovered by genomic and proteomic approaches; however, the functional details and modes of secretion remain unknown for most of them, even when the attention is disclosed to the two SSP families, CLE and PR1-like, important for the development and plant responsiveness to stresses, respectively.

In recent years, the CLE protein family members have been objects of thorough plant developmental studies. It has been found that the CLE peptides regulate stem cell maintenance in the shoot, root meristems, and vascular bundles via the activation of LRR family receptor kinases. For instance, the best studied Arabidopsis CLE member is CLAVATA3 (CLV3), which upon recognition by the CLAVATA1 (CLV1) receptor negatively regulates the expression of the homeodomain transcription factor WUSCHEL, which otherwise promotes stem cell proliferation [rev. in^7^]. The CLE activity is concentrated to a conserved 12–14-amino-acid CLE domain near the C-terminus of the pre-pro-proteins; the signal peptide and pro-protein are processed during transport through the secretory pathway to the apoplast.^8^

CLE peptide orthologs have been identified across plant kingdom; in Arabidopsis, there are 32 identified CLE genes [rev. e. g. in^9,7^]. Besides CLV3, other CLEs have been studied and identified as redundant negative regulators of root meristem activity [e. g. CLE9–13, CLE16–22, CLE27, and CLE45;^10,11,12^]. In roots, CLE9 and 10 negatively regulate xylem periclinal cell division, CLE25 inhibits primary root growth, CLE45 inhibits root phloem development, while CLE41/CLE44 promotes procambium cell division and prevents xylem differentiation [rev. in^9,13^]. For the CLE40, and similarly to members of another related protein family CLE-like (also named GLV from Golven1; CLEL6 and CLEL9), the processing is executed in Golgi by subtilisin proteases [including SBT1.4, SBT1.7, SBT4.13 and SBT6.1;^14–16^]. Besides maturing proteolysis, also inactivating cleavage could occur in the apoplast, which is prevented by further modification by hydroxylation of proline located in the cleavage motif.^15^

The CLE perception is best studied for the CLV3-CLV1 pair; other receptors involved in its perception are CLV2, CORYNE (CRN), RECEPTOR-LIKE PROTEIN KINASE 2 (RPK2), PHLOEM INTERCALATED WITH XYLEM (PXY), PXY-LIKE PXL1 and 2, and related receptor kinases such as BARELY ANY MERISTEM 1 and 2 [BAM1 and BAM2; rev. in^17,7^]. The perception of CLEs is executed via the formation of different multimeric receptor complexes to generate an independent signaling pathway.

The PR1 protein family comprises mostly unknown proteins, that despite high homology and similarity, are not all necessarily involved in the plant defense against pathogens; those that are, by virtue of their RNA expression upregulation, are considered a hallmark of plant immunity activation. The PR1-like encoding genes are highly conserved across kingdoms, including bacteria, evidencing the common origin of this protein [rev. in^18^]. Together, they form a superfamily of secreted proteins with various functions, named CAP [from Cysteine-rich secretory protein [CRISP], Antigen 5, and PR1 proteins;^19^]. The mechanism of action for PR1 is still enigmatic, in contrast to other PR proteins whose function has been elucidated.^20,^^21^ A proteomic/peptidomics approach has revealed that the CAP-derived peptide 1 (CAPE1) from the C-terminus of tomato PR1 protein acts as a damage-associated molecular pattern (DAMP) triggering plant defense responses, such as the burst of reactive oxygen species (ROS) and expression of other PR genes.^22^ Recently, a role for a wheat TaPR1 in the activation of plant defenses dependent on the effective cleavage of its C-terminal motif has been found to be of key importance for the effective defense against *Parastagonospora nodorum*.^23^ However, these plant-specific aspects of the PR1 function may not fully explain its conservation and function throughout kingdoms that may be more related to the PR1 capability to modify the cell death spread.^24–26^

In the genome of A. thaliana, there are 22 PR1-type genes, primarily arranged in clusters of genes [review in ^27^]. Only one of them, A. thaliana PR1 (AtPR1, At2g14610), the most studied member of the family, is activated by pathogens, insects, or chemical treatments, whereas other PR1-type genes are constitutively expressed in roots and pollen.^27^. The AtPR1 trafficking to the extracellular space has been found to be dependent on the presence of RING E3 ligase KEEP-ON-GOING, and vesicular pathway regulators SNARE (BET12, MEMB12, or SYP132) or exocyst complex^[28,29,30,31–33]^. PR1-GFP expressed transiently in *N. benthamiana* leaves exhibits a complex dual trafficking mode, as it is localized in the extracellular space and intracellularly localized in the multivesicular bodies and vacuolar lumen.^26,34^

Other members of the Arabidopsis PR1 protein family remain underexplored so far. The most closely related to AtPR1 is the At2g19990 gene, designated as *PR1-like*.^35^ The At4g33730 has been found to be involved in salt stress tolerance,^36^ while AtPRB1 (At2g14580) gene has been found to be responsive to ethylene and jasmonate treatments.^37^

Despite the fact that the PR1 is used as a marker of systemic defense activation, the mechanistic evidence for its signaling function, or its perception by plant receptors and activation of downstream signaling pathways, is lacking.

In order to uncover the modes of the secretion for multiple members of these two protein families and to verify a secretory pathways differentiation based on a specialization for a cargo type, we speculated that there are specific circumstances which require some family members to be secreted/exocytosed preferentially, and that this can be achieved only by employing accordingly the regulators of vesicular trafficking and exocytosis, mainly similarly over-amplified Rab GTPases, SNAREs, and exocyst subunits.^38–40^ We hypothesize that a direct link between the secreted cargoes and the secretion regulators is establishable using *in silico* RNA co-expression and clustering approaches. Similar approaches have been implemented previously successfully in plants in the identification of components of metabolic pathways [e. g.^41–43^], connecting of the transcriptional regulators and regulated genes networks [e. g.^44,45^], partially also in the search for marker genes for various medical conditions [e. g.^46,47^]. Interestingly, after employing several independent techniques, we discovered that only weak co-expression links could be found for restricted numbers of secreted cargoes and regulators. We propose that there might be particular spatio-temporal requirements for PR1 and CLE proteins to be synthesized and secreted, and efforts to experimentally cover these discrepancies should be invested along with functional studies.

## Material and methods

### Datasets preparation

We prepared gene sets by inquiring Swissprot database and UniprotKB for *A. thaliana* CLE and PR1 proteins, also in concordance with previously published reviews,^18,48,49^, as well as for the vesicular trafficking protein regulators [EXO70s, Q-SNAREs, RABs;^38–40^, Supplementary table I]. The list of analyzed proteins was also extended by proteins reported to be involved in the perception of CLE proteins and pathogen perception [there are no data available on possible PR1 functioning implementing receptors;^48,50^]. The lists of combinations and subsets used for the analyses are provided as well.

The *O. sativa* orthologs dataset for available small secreted proteins PR1-like and CLEs family members, vesicular trafficking regulatory proteins and pathogen perception and CLE receptors was employed for parallel verifying analysis. Thus, five different presets (sheet “presets”) were employed in the search for co-expressional relatedness providing similar results.

### RNA expression analyses

We performed an initial and unrestricted search for correlation using the STRING association network database [https://string-db.org/;^51^] of all PR1-like proteins and CLEs, and all regulator proteins. We employed various data-mined information, using medium and low confidence score (Supplementary Table I, sheet “presets”, column A, C, and I; 0.4 and 0.2, respectively) for network construction, with subsequent application of the maximum clustering similarity (MCS) mode. Visualization of unassigned genes was disabled. Additionally, under the same confidence conditions, the same search modes were performed with a restriction to the co-expression data only.

The gene sets were also subjected to the co-expression pattern analysis using the ATTED-II 11.0 platforms [NetworkDrawer tool;^52,53^ https://atted.jp/], primarily based on microarray experiments (Ath-m.c9 and Ath-m.c4-2). Since these platforms operate with a limitation of entry data up to 100 samples, we prepare protein subsets with exocyst and SNARE regulators with either CLEs and according receptors or PR1-like proteins and related receptors (Supplementary Table 1, sheet “presets”, columns E and G). To partially overcome the constraints of the input dataset, the analyses were performed with the options that allow the extension of the networks by the mutually coexpressed genes or by the inclusion of protein–protein interactors. The visualization of single, unassigned genes was disabled. The obtained networks are visualized by incorporated Cytoscape, showing the existing relationship among genes’ expression by gray lines whose thickness is based on the mutual rank of correlation, which corrects Pearson’s coefficient with regard to the density of genes in correlation space, bold – MR <5, normal – 5 > MR < 30, and thin MR >30).

For further focused analyses, the subset of more tightly coexpressed genes was preselected (selection from STRING analysis, extended by secretion regulators previously found to affect the PR1 trafficking;^26,28,29^) and analyzed in the Genevestigator and Athena platforms. We employed Genevestigator and implemented hierarchical clustering^54^ of preselected cargo, regulators, and receptor genes estimated by Pearson’s coefficient. The clustering of 21 genes was performed out of the RNAseq experiments after treatments with *Pseudomonas* bacteria or flg22 and chitin elicitors (experiments AT-00834 and AT-00742).

The analysis results for the eight genes found to be the most coexpressed with the PR1 in Genevestigator were also verified using on-line available Athena RNA- and protein quantification and clustering tool [Arabidopsis THaliana ExpressioN Atlas; http://athena.proteomics.wzw.tum.de:5002/master_arabidopsis-shiny/;^55^].

Figures obtained out of these platforms and softwares were further modified using Inkscape^56^, available at: https://inkscape.org.

## Results and discussion

### Co-expression in silico analysis of PR1 and CLE families with membrane trafficking regulators

As we documented above, the repertoire of *A. thaliana* SSPs, including PR1-like and CLEs, is often specialized for functioning in selected tissues and/or under specific circumstances. We therefore wanted to investigate if these proteins are secreted by mechanisms controlled by specific regulators/executors of the secretory machinery. The impetus to test this hypothesis stemmed from our recent studies, where we described multilevel, complex trafficking of PR1, which combines classical exocytic pathway with the unconventional secretion via multivesicular bodies and also PR1 retention in the endomembrane system.^26,34^ Since our literature survey revealed that most reports concerning PR1-likes and CLEs do not detail their secretion mechanisms, we employed the *in silico* co-expression analyses of the relatedness of cargo proteins with the regulators of the secretory pathway, as an indication of the functional link. We hypothesized that when there is a need for a specific cargo to be employed in a particular developmental or stress-triggered situation, the SSP cargo and regulators of its transport must be concordantly upregulated. We thus analyzed the co-expression of putative cargo protein subsets PR1-like and CLE proteins with the secretion regulators and machinery proteins such as Rab GTPases, Q-SNARE proteins, and exocyst subunits EXO70 proteins [Supplementary Table I; ^38–40^], as many of these secretion-related genes were previously shown to be transcriptionally regulated in stress responses [e.g. ^57^]. In the case of functional linkage, we would expect to uncover specific clusters consisting of both cargo and regulatory proteins, in relation to certain conditions, which triggered their upregulation. To cover possible expressional non-compliances, we also prepared gene sets comprising the pathogen perception receptors (PRRs) or receptors related to CLE signaling (Supplementary Table I).

**Figure 1.**
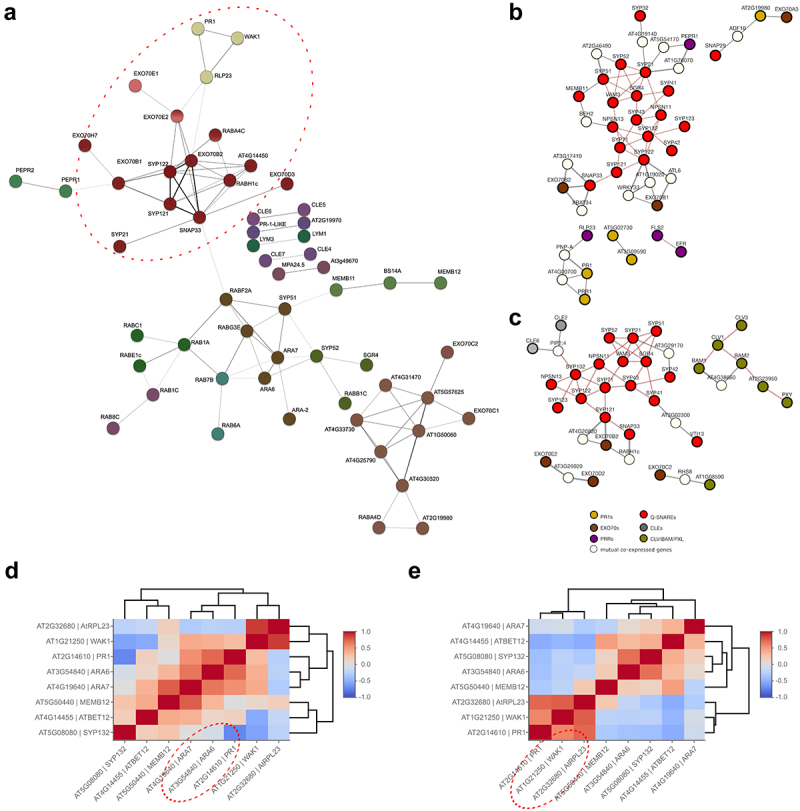
A) Clusters of positively coexpressed genes encoding for PR1-like, CLE proteins, SNARE, Rab GTPases, exocyst EXO70s subunits, and CLE and pathogen perception receptors according to STRING association network (confidence score 0.2) with subsequent MSC mode of clustering; each gene cluster is presented by different color; line thickness connecting node indicates the level of correlation confidence; dotted lines represent connections of clusters. Genes highlighted by the red dotted circles were used in further analyses (Supplementary Figure 2). Co-expression nets of genes encoding for PR1-like (b) and CLE-proteins (c) constructed using the ATTED-II tool (atted.jp/). Color scheme for used protein families: PR-like proteins-yellow, SNARES – red, EXO70s – green, PRRs – violet, CLEs -gray, CLE receptors – olive; the cargoes and regulator genes are all delineated by a bold line; the mutual co-expressed genes are represented by thin-lined white circles; gray lines – co-expression relation (thickness based on mutual rank of correlation which corrects Pearson’s coefficient with regard to the density of genes in correlation space, bold – MR<5, normal – 5> MR<30, and thin MR>30), red lines – protein–protein interactions. (d) Clustering of genes encoding At2g14610 PR1 protein with correlated candidates found in previous analyses, also extended by secretion regulators previously found to affect the PR1 trafficking; the analysis was performed using Athena RNA- (on the left) and protein quantification and clustering tool (on the right).

We subjected prepared gene sets to co-expression pattern analysis using the STRING,^51^ ATTED-II 11.0 platforms [NetworkDrawer tool;^52^], Genevestigator,^54^ and Athena.^55^ First, we performed an unrestricted search for correlation in the STRING database of all PR1-like proteins and CLEs, and all regulator proteins, and from various data-mined information, using a medium confidence score (0.4) for network construction. After the application of the maximum clustering similarity (MCS) mode, we could observe that most of the inspected proteins are indeed related to each other. However, the regulators clustered more tightly with each other, and analogously, some cargoes seem to be more correlated to other cargoes than to regulators (Supplementary Figure 1). When the correlation search was restricted under the same confidence conditions to the co-expression data only, the relatedness between regulators and cargos, as well as within each of the two groups, was below the low confidence threshold level. Interestingly, we observed an analogous clustering pattern with the dataset composed of rice homologs of PR1, PRRs, and secretion regulator families, suggesting the evolutionary conserved relations among these gene families (Supplementary Figure 1). When the co-expression confidence score was decreased to 0.2, and the data set expanded with receptor proteins, some of the clusters were restored, however, again containing mostly either the cargos or the regulators (Figure 1a). Similar results were observed when the ATTED-II platform was employed on protein subsets containing PR1-likes (Figure 1b) and CLEs (Figure 1c), allowing the addition of mutually co-expressed genes, as well as individual protein–protein interactions. Interestingly, the reported presence and processing of CLE proteins within the canonical secretory Rab- and SNARE-regulated pathway, except for CLE2 and 6, may not require synchronization of their gene expression with specific isoforms of regulators.

We were especially interested in the co-expression context of the defense-related PR1 At2g14610, therefore we further investigated the PR1-positive RLP23/WAK1 cluster as obtained by STRING, connected with SNARE/EXO70 cluster, and also expanded by SNAREs and Rabs previously reported to be related with PR1 localization and secretion^26,28,29^ in Genevestigator co-expression analysis. We applied the hierarchical clustering tool with Pearson correlation applied as a distance measure.^54^ We restricted our search to co-expression patterns from the relevant RNAseq experiments performed with treatments by several different Pseudomonas bacteria and elicitors flg22 and chitin. We noticed a more prominent correlation of PR1/RLP23/WAK1 with BET12, SNARE132, ARA6, and ARA7 than with other SNARE and EXO70 participants of defense responses (Supplementary Figure 2). These correlations were further corroborated using Athena RNA and proteomic data exploration tools based on absolute quantification throughout different plant tissues. Using the incorporated correlation and clustering tool, we confirmed the relatedness of gene expression of PR1 with RLP23, WAK1, ARA6/RabF1, and ARA7/RabF2b (Figure 1d). In addition, clustering performed out of the intensity-based absolute quantifications (iBAQ) of each of the included proteins across tissues further highlights the intriguing correlation of PR1 with RLP23 and WAK1, worthy of more elaborated experimental inspection.

Based on these *in silico* analyses, we deduce that despite the limited tendency to be co-expressed under specific conditions, most of the analyzed small soluble protein cargos are not prominently co-expressed with vesicular transport and secretion regulators. There are several possible explanations for this: a) small soluble proteins might be secreted by mechanisms employing other, less canonical participators of secretory pathway regulation; this may be especially relevant in senescence leaf tissue; b) there might be a time lag for small protein expression regulation due to the need for perception, their signaling, post-translational modifications, or a phosphorylation of regulators;^58^ c) similarly to what has been found before – members of amplified gene families show a prominent shift toward positive expression correlations of both transcript and proteins, when compared to randomly selected gene pairs,^55^ which may be a consequence of evolutionary incompleteness of paralogues diversification and randomness in the formation of multimeric complexes; d) there may be so far undetected co-expression analysis software algorithm-caused biases. The validity of these hypotheses might be verified by careful experimental setup and monitoring of cargos´ levels and distributions in appropriate regulators’ knock-out mutants.

## Conclusions

The specific trafficking and secretion of PR1-like and CLE proteins, as examples of small soluble cargo, seem to be regulated in time and tissues by so far unknown mechanisms, as there is no detailed experimental explanation nor *in silico* detectable congruency between co-expression subsets of cargos and secretion regulators. There is only minor and limited overlap in expression patterns achieved with lower stringency of co-expression network buildup, also accomplished by querying with preselected gene candidates rather than the whole gene families. The significance of these findings and the underlying mechanisms for this loose coexpressional relatedness remain to be further tested.

## Supplementary Material

Supplemental Material
