# Supplementary material for: Small secreted proteins and exocytosis regulators: do they go along?
Source: Plant Signal Behav. 2023 Feb 12;18(1):2163340. doi: 10.1080/15592324.2022.2163340 (PMC9930824; doi:10.1080/15592324.2022.2163340)
Supplement: Supplemental Material [file KPSB_A_2163340_SM1517.pdf]

| ***Number*** | ***Sheet name*** | ***Description*** |
|---|---|---|
| 1. | PR1-likes | PR1 protein family |
| 2. | CLEs | CLE protein family |
| 3. | *EXO70s, RABs, Q-SNAREs* | exocytosis regulators |
| 4. | PRR CLE rec. | pathogen recognition receptors (PRRs) and CLE receptors |
| 5 | Rice dataset | *O. sative* homologs of PR1-likes, EXO70s, RABs, Q-SNAREs and PPRs |
| 6. | presets | regulators+cargoes+receptors combinations used for the analyses |

| Gene names | Entry | Protein names | Length |
|---|---|---|---|
| At1g50050 | F4I4X9 | CAP (Cysteine-rich secretory proteins, | 226 |
| At4g25790 | Q9SW04 | At4g25790 (CAP (Cysteine-rich secretory | 210 |
| At5g57625 | Q9FKL1 | At5g57625 (CAP (Cysteine-rich secretory | 207 |
| At5g02730 | Q9LZ12 | CAP (Cysteine-rich secretory proteins, | 205 |
| At4g25780 | Q9SW05 | CAP (Cysteine-rich secretory proteins, | 190 |
| At3g09590 | Q9SF44 | CAP (Cysteine-rich secretory proteins, | 186 |
| At5g66590 | Q9FJY1 | CAP (Cysteine-rich secretory proteins, | 185 |
| At4g31470 | Q9SV22 | CAP (Cysteine-rich secretory proteins, | 185 |
| At2g19970 | Q9SL83 | At2g19970 (CAP (Cysteine-rich secretory | 177 |
| At2g19990 | Q39186 | At2g19990 (Pathogenesis-related | 176 |
| At4g33730 | O81889 | CAP (Cysteine-rich secretory proteins, | 172 |
| At5g26130 | F4JZQ4 | CAP (Cysteine-rich secretory proteins, | 166 |
| At4g33710 | Q39187 | CAP (Cysteine-rich secretory proteins, | 166 |
| At2g19980 | Q9SL82 | CAP (Cysteine-rich secretory proteins, | 165 |
| At4g33720 | O81888 | AT4g33720 (AT4g33720/T16L1_210) | 163 |
| At1g50050 | Q9LPM6 | CAP (Cysteine-rich secretory proteins, | 162 |
| At1g50060 | Q9LPM7 | CAP (Cysteine-rich secretory proteins, | 161 |
| At3g19690 | Q9LJM5 | At3g19690 (CAP (Cysteine-rich secretory | 161 |
| At4g30320 | Q9M0C8 | CAP (Cysteine-rich secretory proteins, | 161 |
| At4g07820 | Q9ZPG2 | At4g07820 (CAP (Cysteine-rich secretory | 160 |
| At2g14580 | Q9ZNS4 | Pathogenesis-related protein 1 (AtPRB1) | 161 |
| At2g14610 | P33154 | Pathogenesis-related protein 1 (PR-1) | 161 |

| Gene names | Entry | Protein names | Length |
|---|---|---|---|
| At2g27250 | Q9XF04 | CLAVATA3 | 96 |
| At1g73165 | Q3ECD6 | CLAVATA3/ESR (CLE)-related protein 1 | 74 |
| At1g69320 | Q4PSX1 | CLAVATA3/ESR (CLE)-related protein 10 | 107 |
| At1g49005 | Q3ECU1 | CLAVATA3/ESR (CLE)-related protein 11 | 99 |
| At1g68795 | Q29PU4 | CLAVATA3/ESR (CLE)-related protein 12 | 118 |
| At1g73965 | Q6NMF0 | CLAVATA3/ESR (CLE)-related protein 13 | 107 |
| At1g63245 | Q3ECJ5 | CLAVATA3/ESR (CLE)-related protein 14 | 80 |
| At2g01505 | Q8S8M2 | CLAVATA3/ESR (CLE)-related protein 16 | 103 |
| At1g70895 | Q8L9H6 | CLAVATA3/ESR (CLE)-related protein 17 | 99 |
| At1g66145 | Q3ECH9 | CLAVATA3/ESR (CLE)-related protein 18 | 101 |
| At3g24225 | Q8W261 | CLAVATA3/ESR (CLE)-related protein 19 | 74 |
| At4g18510 | O49519 | CLAVATA3/ESR (CLE)-related protein 2 | 75 |
| At1g05065 | Q3EDI6 | CLAVATA3/ESR (CLE)-related protein 20 | 83 |
| At5g64800 | Q9LV97 | CLAVATA3/ESR (CLE)-related protein 21 | 106 |
| At5g12235 | Q3E9I4 | CLAVATA3/ESR (CLE)-related protein 22 | 103 |
| At3g28455 | Q8LFL4 | CLAVATA3/ESR (CLE)-related protein 25 | 81 |
| At1g69970 | O04547 | CLAVATA3/ESR (CLE)-related protein 26 | 118 |
| At3g25905 | Q9LUA1 | CLAVATA3/ESR (CLE)-related protein 27 | 91 |
| At1g06225 | Q3EDH8 | CLAVATA3/ESR (CLE)-related protein 3 | 83 |
| At2g31081 | Q8S8N0 | CLAVATA3/ESR (CLE)-related protein 4 | 80 |
| At5g12990 | Q9LXU0 | CLAVATA3/ESR (CLE)-related protein 40 | 80 |
| At3g24770 | Q84W98 | CLAVATA3/ESR (CLE)-related protein 41 | 99 |
| At2g34925 | Q6IWB2 | CLAVATA3/ESR (CLE)-related protein 42 | 88 |
| At1g25425 | Q6IWB1 | CLAVATA3/ESR (CLE)-related protein 43 | 96 |
| At4g13195 | Q941C5 | CLAVATA3/ESR (CLE)-related protein 44 | 112 |
| At1g69588 | Q6IWA9 | CLAVATA3/ESR (CLE)-related protein 45 | 124 |
| At5g59305 | Q8LDN4 | CLAVATA3/ESR (CLE)-related protein 46 | 76 |
| At2g31083 | Q8S8N2 | CLAVATA3/ESR (CLE)-related protein 5 | 81 |
| At2g31085 | Q8S8N3 | CLAVATA3/ESR (CLE)-related protein 6 | 81 |
| At2g31082 | Q8S8N1 | CLAVATA3/ESR (CLE)-related protein 7 | 86 |
| At1g67775 | Q2V4E2 | CLAVATA3/ESR (CLE)-related protein 8 | 86 |
| At1g26600 | Q9FZE4 | CLAVATA3/ESR (CLE)-related protein 9 | 120 |

**EXO70**

| ATGs | Gene names |
|---|---|
| At5g03540 | EXO70A1 |
| At5g52340 | EXO70A2 |
| At5g52350 | EXO70A3 |
| At5g58430 | EXO70B1 |
| At1g07000 | EXO70B2 |
| At5g13150 | EXO70C1 |
| At5g13990 | EXO70C2 |
| At1g72470 | EXO70D1 |
| At1g54090 | EXO70D2 |
| At3g14090 | EXO70D3 |
| At3g29400 | EXO70E1 |
| At5g61010 | EXO70E2 |
| At5g50380 | EXO70F1 |
| At4g31540 | EXO70G1 |
| At1g51640 | EXO70G2 |
| At3g551500 | EXO70H1 |
| At2g39380 | EXO70H2 |
| At3g09530 | EXO70H3 |
| At3g09520 | EXO70H4 |
| At2g28640 | EXO70H5 |
| At1g07725 | EXO70H6 |
| At5g59730 | EXO70H7 |
| At2g28650 | EXO70H8 |

**Q-SNAREs**

| ATGs | Gene names |
|---|---|
| At1g08350 | SYP111, KNOLLE |
| At2g18260 | SYP112 |
| At3g11820 | SYP121, SYR1 |
| At3g52400 | SYP122, SYR4 |
| At4g03330 | SYP123 |
| At1g55410 | SYP124 |
| At1g10980 | SYP125 |
| At3g03800 | SYP131 |
| At5g08080 | SYP132 |
| At5g16830 | SYP21, PEP12 |
| At5g46860 | SYP22, VAM3 |
| At4g17730 | SYP23 |
| At5g05760 | SYP31 |
| At3g24350 | SYP32 |
| At5g26980 | SYP41 |
| At4g02195 | SYP42 |
| At3g05710 | SYP43 |
| At1g47920 | SYP81 |
| AT1G16240 | SYP51 |
| At1g79590 | SYP52 |
| At1g27550 | SYP61 |
| At3g09740 | SYP71 |
| At3g45280 | SYP72 |
| At3g61450 | SYP73 |
| At3g58170 | BET11 |
| At4g14450 | BET12 |
| At1g15590 | GOS11 |
| Atg245200 | GOS12 |
| At2g36900 | MEMB11 |
| At5g50440 | MEMB12 |
| At2g35190 | NPSN11 |
| At1g44640 | NPSN12 |
| At3g17440 | NPSN13 |
| At5g39510 | VTI11, VTI1a |
| At1g25740 | VTI12, VTI1b |
| At3g29100 | VTI13 |
| At5g61210 | SNP11, SNAP33 |
| At5g07880 | SNP12, SNAP29 |
| At1g13530 | SNP13, SNAP30 |

**RABs**

| ATGs | Gene names |
|---|---|
| At1g06400 | RABA1a |
| At1g16920 | RABA1b |
| At5g45750 | RABA1c |
| At4g18800 | RABA1d |
| At4g18430 | RABA1e |
| At5g60860 | RABA1f |
| At3g15060 | RABA1g |
| At2g33870 | RABA1h |
| At1g28550 | RABA1i |
| At1g09630 | RABA2a |
| At1g07410 | RABA2b |
| At3g46830 | RABA2c |
| At5g59150 | RABA2d |
| At1g01200 | RABA3 |
| At5g65270 | RABA4a |
| At4g39990 | RABA4b |
| At5g47960 | RABA4c |
| At3g12160 | RABA4d |
| At2g22390 | RABA4e |
| At5g47520 | RABA5a |
| At3g07410 | RABA5b |
| At2g43130 | RABA5c |
| At2g31680 | RABA5d |
| At1g05810 | RABA5e |
| At1g73640 | RABA6a |
| At1g18200 | RABA6b |
| At4g17160 | RABB1a |
| At4g35860 | RABB1b |
| At4g17170 | RABB1c |
| At1g43890 | RABC1 |
| At5g03530 | RABC2a |
| At3g09910 | RABC2b |
| At3g11730 | RABD1 |
| At1g02130 | RABD2a |
| At5g47200 | RABD2b |
| At4g17530 | RABD2c |
| At3g53610 | RABE1a |
| At4g20360 | RABE1b |
| At3g46060 | RABE1c |
| At5g03520 | RABE1d |
| At3g09900 | RABE1e |
| At3g54840 | RABF1 |
| At5g45130 | RABF2a |
| At4g19640 | RABF2b |
| At5g39620 | RABG1 |
| At2g21880 | RABG2 |
| At4g09720 | RABG3a |
| At1g22740 | RABG3b |
| At3g16100 | RABG3c |
| At1g52280 | RABG3d |
| At1g49300 | RABG3e |
| At3g18820 | RABG3f |
| At5g64990 | RABH1a |
| At2g44610 | RABH1b |
| At4g39890 | RABH1c |
| At2g22290 | RABH1d |
| At5g10260 | RABH1e |

**PRRs**

| ATGs | Gene names |
|---|---|
| At5g46330 | FLS2 |
| At5g20480 | EFR |
| At1g73080 | PEPR1 |
| At1g17750 | PEPR2 |
| At5g60300 | DORN1 |
| At1g61380 | LORE |
| At1g21250 | WAK1 |
| At1g21880 | LYM1 |
| At1g77630 | LYM3 |
| At2g32680 | RLP23 |
| At1g07390 | ReMax/RLP1 |
| At3g05360 | RLP30 |

**CLE receptors**

| ATGs | Gene names |
|---|---|
| At4g18510 | CLE2_ARATH |
| At5g65700 | BAME1_ARATH |
| At5g13290 | CRN_ARATH |
| At3g49670 | BAME2_ARATH |
| At1g75820 | CLV1_ARATH |
| At2g23950 | ser/thre-pk |
| At5g61480 | TDR_ARATH |
| At1g08590 | PXL1_ARATH |
| At4g28650 | MIK1_ARATH |

**Rice PR1-like**

| Gene IDs |
|---|
| Os06g0350600 |
| Os01g0382400 |
| Os10g0191300 |
| Os07g0129200 |
| Os07g0129300 |
| Os01g0382000 |
| Os02g0472500 |
| Os07g0125500 |
| Os07g0124900 |
| Os07g0126301 |
| Os07g0127600 |
| Os07g0125600 |
| Os07g0126401 |
| Os07g0127600 |
| Os02g0786500 |
| Os05g0595200 |
| Os07g0243800 |
| Os07g0125201 |
| Os07g0126100 |
| Os07g0126500 |
| Os07g0127500 |
| Os07g0244100 |
| Os12g0633400 |
| Os02g0786900 |
| Os07g0128800 |
| Os02g0787000 |
| Os07g0128700 |
| Os04g0289700 |
| Os04g0289600 |
| Os07g0127800 |
| Os07g0127700 |
| Os07g0127900 |
| Os04g0289500 |
| Os05g0595000 |
| Os04g0290500 |
| Os03g0733332 |

**rice EXO70s**

| Gene IDs |
|---|
| Os02g0149700 |
| Os06g0698600 |
| Os11g0157400 |
| Os04g0685600 |
| Os04g0685500 |
| Os01g0921400 |
| Os04g0382200 |
| Os12g0165600 |
| Os09g0439600 |
| Os02g0505400 |
| Os11g0167600 |
| Os08g0455700 |
| Os08g0530300 |
| Os01g0763700 |
| Os01g0763750 |
| Os05g0473500 |
| Os01g0827500 |
| Os06g0255900 |
| Os08g0519900 |
| Os01g0827600 |
| Os10g0140100 |
| Os07g0210000 |
| Os02g0575900 |
| Os11g0100800 |
| Os12g0100700 |
| Os01g0383100 |
| Os01g0905300 |
| Os05g0369500 |
| Os06g0183600 |
| Os05g0369300 |
| Os05g0369700 |
| Os03g0448200 |
| Os07g0210900 |
| Os01g0768032 |
| Os01g0905200 |
| Os04g0111500 |
| Os08g0232700 |
| Os07g0210100 |
| Os11g0649900 |
| Os11g0649900 |
| Os07g0210300 |
| Os07g0210675 |
| Os07g0211000 |
| Os09g0347300 |
| Os03g0358600 |

**rice Q-SNAREs**

| Gene IDs | Gene names |
|---|---|
| Os03g0736500 | OsSyp111 |
| Os12g0192300 | OsSyp112 |
| Os03g0787000 | OsSyp121 |
| Os02g0209900 | OsSyp124 |
| Os06g0590500 | OsSyp125 |
| Os07g0164300 | OsSyp131 |
| Os06g0168500 | OsSyp132 |
| Os01g0254900 | OsSyp21 |
| Os06g0223000 | OsSyp22 |
| Os02g0702800 | OsSyp23 |
| Os01g0179200 | OsSyp32 |
| Os06g0116300 | OsSyp42 |
| Os12g0154400 | OsSyp81 |
| Os04g0530400 | OsSyp82 |
| Os08g0277900 | OsSyp52 |
| Os02g0119400 | OsSyp51 |
| Os01g0963300 | OsSyp61 |
| Os11g0168100 | OsSyp62 |
| Os08g0244100 | OsSyp63 |
| Os05g0553700 | OsSyp71 |
| Os09g0359700 | OsSyp72 |
| Os02g0820700 | OsBet11 |
| Os08g0563300 | OsBet12 |
| Os08g0440000 | OsGos11 |
| Os02g0126800 | OsGos12 |
| Os09g0416700 | OsGos13 |
| Os03g0655200 | OsMemb12 |
| Os06g0715100 | OsNpsn11 |
| Os07g0637400 | OsNpsn12 |
| Os03g0369800 | OsNpsn13 |
| Os01g0560200 | OsVti11 |
| Os01g0707300 | OsVti12 |
| Os02g0437200 | OsSNAP11 |
| Os03g0212400 | OsSNAP12 |
| Os02g0529500 | OsSNAP13 |

**rice RABs**

| Gene IDs | Gene names |
|---|---|
| Os05g010520 | OsRab1A1 |
| Os01g0558600 | OsRab1B2 |
| Os01g0179700 | OsRab1C1 |
| Os02g0658100 | OsRab1C2 |
| Os02g0653800 | OsRab1C3 |
| Os04g0470100 | OsRab2A1 |
| Os02g0586400 | OsRab2A2 |
| Os10g0208800 | OsRab2B1 |
| Os10g0208200 | OsRab2B2 |
| Os03g0151900 | OsRab5A1 |
| Os10g0441800 | OsRab5A2 |
| Os03g0666500 | OsRab5B1 |
| Os12g0631100 | OsRab5B2 |
| Os06g0687100 | OsRab5C1 |
| Os05g0341600 | OsRab5D1 |
| Os07g0496000 | OsRab6A1 |
| Os03g0191400 | OsRab6B2a |
| Os05g0536900 | OsRab7A1 |
| Os01g0714900 | OsRab7A2 |
| Os01g0227300 | OsRab7B1 |
| Os05g0516600 | OsRab7B3 |
| Os05g0461300 | OsRab8A2 |
| Os07g0239400 | OsRab8A3 |
| Os03g0819900 | OsRab8A5 |
| Os07g0195100 | OsRab8B1 |
| Os03g0843100 | OsRab11A1 |
| Os05g0105100 | OsRab11A2 |
| Os05g0280200 | OsRab11B1 |
| Os03g0823700 | OsRab11B2 |
| Os01g0848700 | OsRab11B3 |
| Os01g0667600 | OsRab11C1 |
| Os09g0327100 | OsRab11C2 |
| Os05g0564400 | OsRab11C3 |
| Os06g0551400 | OsRab11D1 |
| Os01g0750000 | OsRab11D2 |
| Os05g0516800 | OsRab11D3 |
| Os10g0377400 | OsRab11E1 |
| Os09g0281700 | OsRab11E2 |
| Os06g0714600 | OsRab11F1 |
| Os09g0527600 | OsRab11F2 |
| Os07g0634200 | OsRab11G1 |
| Os08g0525000 | OsRab11G2 |
| Os10g0456600 | OsRab18B1 |
| Os03g0146000 | OsRab18B2 |

**rice PRRs receptors homologs**

| Gene IDs | Gene names |
|---|---|
| Os04g0618700 | OsFLS2 |
| Os12g0620000 | EFR |
| Os08g0446200 | PEPR-like |
| Os08g0446301 | |
| Os08g0446400 | |
| Os10g0155800 | |
| Os09g0471800 | WAK1-like |
| Os04g0367900 | |
| Os01g0364800 | |
| Os02g0808300 | |
| Os12g0614800 | |
| Os12g0614851 | |
| Os02g0811200 | |
| Os02g0807900 | |
| Os06g0705200 | |
| Os01g0364100 | |
| Os09g0373800 | |
| Os09g0471500 | |

| all cargo+reg. |
|---|
| At5g03540 |
| At5g52340 |
| At5g52350 |
| At5g58430 |
| At1g07000 |
| At5g13150 |
| At5g13990 |
| At1g72470 |
| At1g54090 |
| At3g14090 |
| At3g29400 |
| At5g61010 |
| At5g50380 |
| At4g31540 |
| At1g51640 |
| At3g551500 |
| At2g39380 |
| At3g09530 |
| At3g09520 |
| At2g28640 |
| At1g07725 |
| At5g59730 |
| At2g28650 |
| At1g08350 |
| At2g18260 |
| At3g11820 |
| At3g52400 |
| At4g03330 |
| At1g55410 |
| At1g10980 |
| At3g03800 |
| At5g08080 |
| At5g16830 |
| At5g46860 |
| At4g17730 |
| At5g05760 |
| At3g24350 |
| At5g26980 |
| At4g02195 |
| At3g05710 |
| At1g47920 |
| AT1G16240 |
| At1g79590 |
| At1g27550 |
| At3g09740 |
| At3g45280 |
| At3g61450 |
| At3g58170 |
| At4g14450 |
| At1g15590 |
| Atg245200 |
| At2g36900 |
| At5g50440 |
| At2g35190 |
| At1g44640 |
| At3g17440 |
| At5g39510 |
| At1g25740 |
| At3g29100 |
| At5g61210 |
| At5g07880 |
| At1g13530 |
| At1g06400 |
| At1g16920 |
| At5g45750 |
| At4g18800 |
| At4g18430 |
| At5g60860 |
| At3g15060 |
| At2g33870 |
| At1g28550 |
| At1g09630 |
| At1g07410 |
| At3g46830 |
| At5g59150 |
| At1g01200 |
| At5g65270 |
| At4g39990 |
| At5g47960 |
| At3g12160 |
| At2g22390 |
| At5g47520 |
| At3g07410 |
| At2g43130 |
| At2g31680 |
| At1g05810 |
| At1g73640 |
| At1g18200 |
| At4g17160 |
| At4g35860 |
| At4g17170 |
| At1g43890 |
| At5g03530 |
| At3g09910 |
| At3g11730 |
| At1g02130 |
| At5g47200 |
| At4g17530 |
| At3g53610 |
| At4g20360 |
| At3g46060 |
| At5g03520 |
| At3g09900 |
| At3g54840 |
| At5g45130 |
| At4g19640 |
| At5g39620 |
| At2g21880 |
| At4g09720 |
| At1g22740 |
| At3g16100 |
| At1g52280 |
| At1g49300 |
| At3g18820 |
| At5g64990 |
| At2g44610 |
| At4g39890 |
| At2g22290 |
| At5g10260 |
| At2g27250 |
| At1g73165 |
| At1g69320 |
| At1g49005 |
| At1g68795 |
| At1g73965 |
| At1g63245 |
| At2g01505 |
| At1g70895 |
| At1g66145 |
| At3g24225 |
| At4g18510 |
| At1g05065 |
| At5g64800 |
| At5g12235 |
| At3g28455 |
| At1g69970 |
| At3g25905 |
| At1g06225 |
| At2g31081 |
| At5g12990 |
| At3g24770 |
| At2g34925 |
| At1g25425 |
| At4g13195 |
| At1g69588 |
| At5g59305 |
| At2g31083 |
| At2g31085 |
| At2g31082 |
| At1g67775 |
| At1g26600 |
| At1g50050 |
| At4g25790 |
| At5g57625 |
| At5g02730 |
| At4g25780 |
| At3g09590 |
| At5g66590 |
| At4g31470 |
| At2g19970 |
| At2g19990 |
| At4g33730 |
| At5g26130 |
| At4g33710 |
| At2g19980 |
| At4g33720 |
| At1g50050 |
| At1g50060 |
| At3g19690 |
| At4g30320 |
| At4g07820 |
| At2g14580 |
| At2g14610 |

| all cargo+reg.+recep. |
|---|
| At5g03540 |
| At5g52340 |
| At5g52350 |
| At5g58430 |
| At1g07000 |
| At5g13150 |
| At5g13990 |
| At1g72470 |
| At1g54090 |
| At3g14090 |
| At3g29400 |
| At5g61010 |
| At5g50380 |
| At4g31540 |
| At1g51640 |
| At3g551500 |
| At2g39380 |
| At3g09530 |
| At3g09520 |
| At2g28640 |
| At1g07725 |
| At5g59730 |
| At2g28650 |
| At1g08350 |
| At2g18260 |
| At3g11820 |
| At3g52400 |
| At4g03330 |
| At1g55410 |
| At1g10980 |
| At3g03800 |
| At5g08080 |
| At5g16830 |
| At5g46860 |
| At4g17730 |
| At5g05760 |
| At3g24350 |
| At5g26980 |
| At4g02195 |
| At3g05710 |
| At1g47920 |
| AT1G16240 |
| At1g79590 |
| At1g27550 |
| At3g09740 |
| At3g45280 |
| At3g61450 |
| At3g58170 |
| At4g14450 |
| At1g15590 |
| Atg245200 |
| At2g36900 |
| At5g50440 |
| At2g35190 |
| At1g44640 |
| At3g17440 |
| At5g39510 |
| At1g25740 |
| At3g29100 |
| At5g61210 |
| At5g07880 |
| At1g13530 |
| At1g06400 |
| At1g16920 |
| At5g45750 |
| At4g18800 |
| At4g18430 |
| At5g60860 |
| At3g15060 |
| At2g33870 |
| At1g28550 |
| At1g09630 |
| At1g07410 |
| At3g46830 |
| At5g59150 |
| At1g01200 |
| At5g65270 |
| At4g39990 |
| At5g47960 |
| At3g12160 |
| At2g22390 |
| At5g47520 |
| At3g07410 |
| At2g43130 |
| At2g31680 |
| At1g05810 |
| At1g73640 |
| At1g18200 |
| At4g17160 |
| At4g35860 |
| At4g17170 |
| At1g43890 |
| At5g03530 |
| At3g09910 |
| At3g11730 |
| At1g02130 |
| At5g47200 |
| At4g17530 |
| At3g53610 |
| At4g20360 |
| At3g46060 |
| At5g03520 |
| At3g09900 |
| At3g54840 |
| At5g45130 |
| At4g19640 |
| At5g39620 |
| At2g21880 |
| At4g09720 |
| At1g22740 |
| At3g16100 |
| At1g52280 |
| At1g49300 |
| At3g18820 |
| At5g64990 |
| At2g44610 |
| At4g39890 |
| At2g22290 |
| At5g10260 |
| At2g27250 |
| At1g73165 |
| At1g69320 |
| At1g49005 |
| At1g68795 |
| At1g73965 |
| At1g63245 |
| At2g01505 |
| At1g70895 |
| At1g66145 |
| At3g24225 |
| At4g18510 |
| At1g05065 |
| At5g64800 |
| At5g12235 |
| At3g28455 |
| At1g69970 |
| At3g25905 |
| At1g06225 |
| At2g31081 |
| At5g12990 |
| At3g24770 |
| At2g34925 |
| At1g25425 |
| At4g13195 |
| At1g69588 |
| At5g59305 |
| At2g31083 |
| At2g31085 |
| At2g31082 |
| At1g67775 |
| At1g26600 |
| At1g50050 |
| At4g25790 |
| At5g57625 |
| At5g02730 |
| At4g25780 |
| At3g09590 |
| At5g66590 |
| At4g31470 |
| At2g19970 |
| At2g19990 |
| At4g33730 |
| At5g26130 |
| At4g33710 |
| At2g19980 |
| At4g33720 |
| At1g50050 |
| At1g50060 |
| At3g19690 |
| At4g30320 |
| At4g07820 |
| At2g14580 |
| At2g14610 |
| At5g46330 |
| At5g20480 |
| At1g73080 |
| At1g17750 |
| At5g60300 |
| At1g61380 |
| At1g21250 |
| At1g21880 |
| At1g77630 |
| At2g32680 |
| At1g07390 |
| At3g05360 |
| At4g18510 |
| At5g65700 |
| At5g13290 |
| At3g49670 |
| At1g75820 |
| At2g23950 |
| At5g61480 |
| At1g08590 |
| At4g28650 |

| CLEs+SNARES+EXO70+CLE rec. |
|---|
| At5g03540 |
| At5g52340 |
| At5g52350 |
| At5g58430 |
| At1g07000 |
| At5g13150 |
| At5g13990 |
| At1g72470 |
| At1g54090 |
| At3g14090 |
| At3g29400 |
| At5g61010 |
| At5g50380 |
| At4g31540 |
| At1g51640 |
| At3g551500 |
| At2g39380 |
| At3g09530 |
| At3g09520 |
| At2g28640 |
| At1g07725 |
| At5g59730 |
| At2g28650 |
| At1g08350 |
| At2g18260 |
| At3g11820 |
| At3g52400 |
| At4g03330 |
| At1g55410 |
| At1g10980 |
| At3g03800 |
| At5g08080 |
| At5g16830 |
| At5g46860 |
| At4g17730 |
| At5g05760 |
| At3g24350 |
| At5g26980 |
| At4g02195 |
| At3g05710 |
| At1g47920 |
| AT1G16240 |
| At1g79590 |
| At1g27550 |
| At3g09740 |
| At3g45280 |
| At3g61450 |
| At3g58170 |
| At4g14450 |
| At1g15590 |
| Atg245200 |
| At2g36900 |
| At5g50440 |
| At2g35190 |
| At1g44640 |
| At3g17440 |
| At5g39510 |
| At1g25740 |
| At3g29100 |
| At5g61210 |
| At5g07880 |
| At1g13530 |
| At2g27250 |
| At1g73165 |
| At1g69320 |
| At1g49005 |
| At1g68795 |
| At1g73965 |
| At1g63245 |
| At2g01505 |
| At1g70895 |
| At1g66145 |
| At3g24225 |
| At4g18510 |
| At1g05065 |
| At5g64800 |
| At5g12235 |
| At3g28455 |
| At1g69970 |
| At3g25905 |
| At1g06225 |
| At2g31081 |
| At5g12990 |
| At3g24770 |
| At2g34925 |
| At1g25425 |
| At4g13195 |
| At1g69588 |
| At5g59305 |
| At2g31083 |
| At2g31085 |
| At2g31082 |
| At1g67775 |
| At1g26600 |
| At4g18510 |
| At5g65700 |
| At5g13290 |
| At3g49670 |
| At1g75820 |
| At2g23950 |
| At5g61480 |
| At1g08590 |
| At4g28650 |

| PR1-l.s+SNARES+EXO70+PRR rec. |
|---|
| At5g03540 |
| At5g52340 |
| At5g52350 |
| At5g58430 |
| At1g07000 |
| At5g13150 |
| At5g13990 |
| At1g72470 |
| At1g54090 |
| At3g14090 |
| At3g29400 |
| At5g61010 |
| At5g50380 |
| At4g31540 |
| At1g51640 |
| At3g551500 |
| At2g39380 |
| At3g09530 |
| At3g09520 |
| At2g28640 |
| At1g07725 |
| At5g59730 |
| At2g28650 |
| At1g08350 |
| At2g18260 |
| At3g11820 |
| At3g52400 |
| At4g03330 |
| At1g55410 |
| At1g10980 |
| At3g03800 |
| At5g08080 |
| At5g16830 |
| At5g46860 |
| At4g17730 |
| At5g05760 |
| At3g24350 |
| At5g26980 |
| At4g02195 |
| At3g05710 |
| At1g47920 |
| AT1G16240 |
| At1g79590 |
| At1g27550 |
| At3g09740 |
| At3g45280 |
| At3g61450 |
| At3g58170 |
| At4g14450 |
| At1g15590 |
| Atg245200 |
| At2g36900 |
| At5g50440 |
| At2g35190 |
| At1g44640 |
| At3g17440 |
| At5g39510 |
| At1g25740 |
| At3g29100 |
| At5g61210 |
| At5g07880 |
| At1g13530 |
| At1g50050 |
| At4g25790 |
| At5g57625 |
| At5g02730 |
| At4g25780 |
| At3g09590 |
| At5g66590 |
| At4g31470 |
| At2g19970 |
| At2g19990 |
| At4g33730 |
| At5g26130 |
| At4g33710 |
| At2g19980 |
| At4g33720 |
| At1g50050 |
| At1g50060 |
| At3g19690 |
| At4g30320 |
| At4g07820 |
| At2g14580 |
| At2g14610 |
| At5g46330 |
| At5g20480 |
| At1g73080 |
| At1g17750 |
| At5g60300 |
| At1g61380 |
| At1g21250 |
| At1g21880 |
| At1g77630 |
| At2g32680 |
| At1g07390 |
| At3g05360 |

| Rice PR1-l.s+reg.+PRR rec. |
|---|
| Os02g0149700 |
| Os06g0698600 |
| Os11g0157400 |
| Os04g0685600 |
| Os04g0685500 |
| Os01g0921400 |
| Os04g0382200 |
| Os12g0165600 |
| Os09g0439600 |
| Os02g0505400 |
| Os11g0167600 |
| Os08g0455700 |
| Os08g0530300 |
| Os01g0763700 |
| Os01g0763750 |
| Os05g0473500 |
| Os01g0827500 |
| Os06g0255900 |
| Os08g0519900 |
| Os01g0827600 |
| Os10g0140100 |
| Os07g0210000 |
| Os02g0575900 |
| Os11g0100800 |
| Os12g0100700 |
| Os01g0383100 |
| Os01g0905300 |
| Os05g0369500 |
| Os06g0183600 |
| Os05g0369300 |
| Os05g0369700 |
| Os03g0448200 |
| Os07g0210900 |
| Os01g0768032 |
| Os01g0905200 |
| Os04g0111500 |
| Os08g0232700 |
| Os07g0210100 |
| Os11g0649900 |
| Os11g0649900 |
| Os07g0210300 |
| Os07g0210675 |
| Os07g0211000 |
| Os09g0347300 |
| Os03g0358600 |
| Os03g0736500 |
| Os12g0192300 |
| Os03g0787000 |
| Os02g0209900 |
| Os06g0590500 |
| Os07g0164300 |
| Os06g0168500 |
| Os01g0254900 |
| Os06g0223000 |
| Os02g0702800 |
| Os01g0179200 |
| Os06g0116300 |
| Os12g0154400 |
| Os04g0530400 |
| Os08g0277900 |
| Os02g0119400 |
| Os01g0963300 |
| Os11g0168100 |
| Os08g0244100 |
| Os05g0553700 |
| Os09g0359700 |
| Os02g0820700 |
| Os08g0563300 |
| Os08g0440000 |
| Os02g0126800 |
| Os09g0416700 |
| Os03g0655200 |
| Os06g0715100 |
| Os07g0637400 |
| Os03g0369800 |
| Os01g0560200 |
| Os01g0707300 |
| Os02g0437200 |
| Os03g0212400 |
| Os02g0529500 |
| Os05g010520 |
| Os01g0558600 |
| Os01g0179700 |
| Os02g0658100 |
| Os02g0653800 |
| Os04g0470100 |
| Os02g0586400 |
| Os10g0208800 |
| Os10g0208200 |
| Os03g0151900 |
| Os10g0441800 |
| Os03g0666500 |
| Os12g0631100 |
| Os06g0687100 |
| Os05g0341600 |
| Os07g0496000 |
| Os03g0191400 |
| Os05g0536900 |
| Os01g0714900 |
| Os01g0227300 |
| Os05g0516600 |
| Os05g0461300 |
| Os07g0239400 |
| Os03g0819900 |
| Os07g0195100 |
| Os03g0843100 |
| Os05g0105100 |
| Os05g0280200 |
| Os03g0823700 |
| Os01g0848700 |
| Os01g0667600 |
| Os09g0327100 |
| Os05g0564400 |
| Os06g0551400 |
| Os01g0750000 |
| Os05g0516800 |
| Os10g0377400 |
| Os09g0281700 |
| Os06g0714600 |
| Os09g0527600 |
| Os07g0634200 |
| Os08g0525000 |
| Os10g0456600 |
| Os03g0146000 |
| Os06g0350600 |
| Os01g0382400 |
| Os10g0191300 |
| Os07g0129200 |
| Os07g0129300 |
| Os01g0382000 |
| Os02g0472500 |
| Os07g0125500 |
| Os07g0124900 |
| Os07g0126301 |
| Os07g0127600 |
| Os07g0125600 |
| Os07g0126401 |
| Os07g0127600 |
| Os02g0786500 |
| Os05g0595200 |
| Os07g0243800 |
| Os07g0125201 |
| Os07g0126100 |
| Os07g0126500 |
| Os07g0127500 |
| Os07g0244100 |
| Os12g0633400 |
| Os02g0786900 |
| Os07g0128800 |
| Os02g0787000 |
| Os07g0128700 |
| Os04g0289700 |
| Os04g0289600 |
| Os07g0127800 |
| Os07g0127700 |
| Os07g0127900 |
| Os04g0289500 |
| Os05g0595000 |
| Os04g0290500 |
| Os03g0733332 |
| Os04g0618700 |
| Os12g0620000 |
| Os08g0446200 |
| Os08g0446301 |
| Os08g0446400 |
| Os10g0155800 |
| Os09g0471800 |
| Os04g0367900 |
| Os01g0364800 |
| Os02g0808300 |
| Os12g0614800 |
| Os12g0614851 |
| Os02g0811200 |
| Os02g0807900 |
| Os06g0705200 |
| Os01g0364100 |
| Os09g0373800 |
| Os09g0471500 |
